# Islamic fasting and multiple sclerosis

**DOI:** 10.1186/1471-2377-14-56

**Published:** 2014-03-22

**Authors:** Soodeh Razeghi Jahromi, Mohammad Ali Sahraian, Fereshteh Ashtari, Hormoz Ayromlou, Massoud Etemadifar, Majid Ghaffarpour, Ehsan Mohammadianinejad, Shahriar Nafissi, Alireza Nickseresht, Vahid Shaygannejad, Mansoreh Togha, Hamid Reza Torabi, Shadi Ziaie

**Affiliations:** 1 MS Research Center, Neuroscience Institute, Sina Hospital, Tehran University of Medical Sciences, Tehran, Iran; 2 Isfahan Neuroscience Research Center, Isfahan University of Medical Sciences, Isfahan, Iran; 3 Department of Neurology, Faculty of Medicine, Tabriz University of Medical Sciences, Tabriz, Iran; 4 Iranian Center for Neurological Research, Imam Khomini Hospital, Tehran University of Medical Sciences, Tehran, Iran; 5 Department of Neurology, Golestan Hospital, Ahwaz University of Medical Sciences, Ahwaz, Iran; 6 Department of Neurology, Shariati Hospital, Tehran University of Medical Sciences, Tehran, Iran; 7 Department of Neurology, Namazi Hospital, Shiraz University of Medical Sciences, Shiraz, Iran; 8 Department of Neurology, Kashani Hospital, Isfahan University of Medical Sciences, Isfahan, Iran; 9 Jam Hospital, Iranian MS Society, Tehran, Iran; 10 Department of Clinical Pharmacy, School of Pharmacy, Shahid Beheshti University of Medical Sciences, Tehran, Iran; 11 Iran MS and Neuroimmunology Research Center, Isfahan University of Medical Sciences, Isfahan, Iran

**Keywords:** Ramadan fasting, Multiple sclerosis, Calorie restriction

## Abstract

**Background:**

Month-long daytime Ramadan fasting pose s major challenges to multiple sclerosis (MS) patients in Muslim countries. Physicians should have practical knowledge on the implications of fasting on MS. We present a summary of database searches (Cochrane Database of Systematic Reviews, PubMed) and a mini-symposium on Ramadan fasting and MS. In this symposium, we aimed to review the effect of fasting on MS and suggest practical guidelines on management.

**Discussion:**

In general, fasting is possible for most stable patients. Appropriate amendment of drug regimens, careful monitoring of symptoms, as well as providing patients with available evidence on fasting and MS are important parts of management. Evidence from experimental studies suggests that calorie restriction before disease induction reduces inflammation and subsequent demyelination and attenuates disease severity. Fasting does not appear to have unfavorable effects on disease course in patients with mild disability (Expanded Disability Status Scale (EDSS) score ≤3). Most experts believed that during fasting (especially in summer), some MS symptoms (fatigue, fatigue perception, dizziness, spasticity, cognitive problems, weakness, vision, balance, gait) might worsen but return to normal levels during feasting. There was a general consensus that fasting is not safe for patients: on high doses of anti-convulsants, anti-spastics, and corticosteroids; with coagulopathy or active disease; during attacks; with EDSS score ≥7.

**Summary:**

These data suggest that MS patients should have tailored care. Fasting in MS patients is a challenge that is directly associated with the spiritual belief of the patient.

## Background

Several religions mention periods of fasting in their respective holy books. A demographic study in 2009 reported that 23% (1.57 billion) of the world population is Muslim [1]. This percentage is growing by ≈3% per year. Fasting during the holy month of Ramadan is considered to be one of the five pillars of Islam. During Ramadan, Muslims are forbidden from eating, drinking, smoking, and sexual congress from dusk to dawn. Travelers, women who are menstruating, pregnant or lactating, children as well as the sick and disabled are exempt from fasting [2]. However, many Muslims who are eligible for exemption (including individuals with mild, moderate, and severe medical conditions) choose to fast. Moreover, recent evidence suggests that there is no contraindication to Islamic fasting for patients with mild coronary artery disease, valvular problems, stable asthma, type-2 diabetes mellitus (T2DM), asymptomatic peptic ulcers, and intestinal motility disorders. Consequently, Muslim patients with various medical disorders are seeking advice about the safety and feasibility of fasting [3].

Multiple sclerosis (MS) is one of these medical conditions. MS is a chronic demyelinating disease with an unclear etiology. It affects≈2.5 million individuals worldwide and has been identified as the most common debilitating neurological disease in young adults [4]. MS is an immune-mediated inflammatory disease of the central nervous system (CNS) that, in the most severe cases, can lead to irreversible disability [5].

The concern for Muslim MS patients is whether Ramadan fasting might have an unfavorable effect on disease course. This debate became more heated after the work of Esquifino et al. on the animal model of MS: experimental autoimmune encephalomyelitis (EAE). In studies in humans and animals, the effect of fasting on health is commonly studied in three forms: calorie restriction (CR), alternate-day fasting (ADF) and dietary restriction. ADF and CR can induce similar changes upon metabolism and physiology. Esquifino et al. reported that CR improves signs of EAE [6]. Hence, we reviewed the opinions of neurologists involved in the care of MS patients with regard to the clinical impact of Ramadan fasting on MS irrespective of their religious beliefs.

For this purpose, we first reviewed current evidence regarding fasting and MS. Second, we prepared a concise summary of the points discussed in a mini-symposium on the effect of fasting on MS patients. In this symposium, we asked neurologists, nutritionists, and pharmacists to present a summary of available evidence as well as their clinical experiences of fasting and MS. Consequently, we aimed to provide practical guidance for MS patients who wished to fast with respect to general health, disability score, level of activity, sex, weight, symptoms and employment.

Information regarding Ramadan fasting and MS was obtained through the Cochrane Database of Systematic Reviews and PubMed databases. Relevant abstracted reports were considered from initiation of these databases to June 2013by combining the term “Ramadan” (476 entries) with terms specific to each of the topics under investigation. All relevant abstracts on a “ketogenic diet” (703 items) as well as “calorie restriction” or “fasting” combined with the terms “neuroprotection” and” immunity” (38 items) were reviewed. The results of the mini-symposium were recorded.

## Discussion

### Fasting in animal studies

CR with nutritional maintenance has been proposed to extend lifespan in fruit fly, nematodes, zebra fish, spiders, rodents, and rotifers [7]. In addition to slowing the aging process, CR has been demonstrated to delay the onset of atherosclerosis, cardiomyopathies, renal disease, T2DM, cancers, and respiratory disease [7].

With regard to neurological diseases and fasting, CR represents the first effective treatment for epilepsy in medical history [7]. CR results in an increase in the serum level of the ketone bodyβ-hydroxybutyrate. This rise in β-hydroxybutyrate level correlates with a significant reduction in the vulnerability of hippocampal neurons to injection of the mineral salt kainite. In the 1920s, the ketogenic characteristics of CR led to the development of the high-fat, low-carbohydrate ketogenic diet (KD) [7]. The antiepileptic properties of KD could be explained (at least in part)by its anti-apoptotic and antioxidative properties. Recent studies suggested that the pro-apoptotic proteinnuclear clusterindid not accumulate in the hippocampi of KD-fed mice in which seizures were induced by kainic acid (KA) [8]. KD also reduced KA-induced cell death in the hippocampus by reducing caspase-3 levels and blocking the dissociation of Bcl-2-associated death proteinfrom 14-3-3 proteins [9]. Noh et al. undertook an *invitro* study on the effect of acetoacetate (AA) on glutamate cytotoxicity in primary hippocampal neurons in rats and the mouse hippocampus cell line HT22. Pretreatment with AA reduced production of reactive oxygen species in the HT22 cell line. AA also decreased the appearance of propidiumiodine-positive and annexin V-positive HT22 cells, which are representative of necrosis and apoptosis, respectively [10].

Accumulating data suggest that the beneficial effects of CR are not limited to ketogenic properties. CR *per se* has profound neuroprotective effects in animal models relevant to the pathogenesis of neurodegenerative disorders such as Huntington disease, Parkinson disease, Alzheimer disease and stroke. Opalachet al. investigated the effect of 40% lifelong CR on age-related oxidative damage in peripheral nerves. They found that CR ameliorates the age-related accumulation of crosslinked and oxidized substances in peripheral nerves. CR also attenuated age-related increments in levels of nuclear factor kappaB (NF-κB), phospho-IkB, and tumor necrosis factor (TNF)-α [11]. Age-related oxidation of polyunsaturated fatty acids in myelin results in the formation of hydroxyalkenals and hydroperoxidases such as4-hydroxynonenal (HNE) and malondialdehyde (MDA), which react with proteins and change their surface hydrophobicity. CR suppresses age-related increases in the MDA-adducted proteins of sciatic nerves. CR also reduces HNE-positive areas throughout axonal and glial compartments [12]. Several other molecular and cellular mechanisms have also been proposed for the neuroprotective action of CR: decrease in the mitochondrial production of free radicals [13]; promotion of antioxidant defense [7]; induction of bioenergetic compounds [7]; elevation in the activity of sirtuin [14]; rise in the activity of neurotrophic factors [15]; increase in the activity of protein chaperones [7]; enhancement of neurogenesis [7]; imposition of anti-inflammatory properties [7]; reduction in leptin levels [16]; and reduction of the apoptosis of oligodendrocytes [15]. Each of these molecular and cellular mechanisms makes CR beneficial by protecting against MS and EAE.

Only three studies on CR in an animal model of MS (EAE) have been carried out. In two of these studies, Esquifino et al. found that restricting energy intake by 33% or 66% from 15 days before EAE induction decrease disease severity in rats [6,17]. Rats in the latter group were totally protected against EAE. Subjecting rats to 66% CR led to impaired proliferation of lymphocytes, reduction in the number of CD4+ cells in lymphoid organs, and suppressed production of interferon (IFN)-γ. In a study by Piccio et al., mice were subjected to 40% CR from 5 weeks before the induction of disease. They observed comparable beneficial effects on disease course [18]. They found that CR worked through enhancement of the production of endogenous glucocorticoids but not through suppression of the immune system. In the three studies mentioned above, three concepts were proposed for the beneficial effects of CR.

### Concept 1: improving immune function

Long-term CR improves T-cell function and delays immune senescence. According to a study by Squifino et al., 66% CR results in modification of the 24-hrhythmicity of lymph nodes infected by tuberculosis, T cells, functions of CD4 + -CD8+ and CD4+ cell subsets, as well as mitogenic responses in lymph nodes to concanavalin A and lipopolysaccharide. Furthermore, mean values of the submaximal lymph-node response to concanavalin A and number of CD4+ cells increased. Conversely, the number of B-cells and secretion of IFN-γ decreased [17]. In the study by Piccio et al., 40% CR ameliorated the signs of EAE without suppression of proliferation of T cells. Long-term CR reduced susceptibility to infectious diseases by enhancing T-cell function [18]. Taken together, application of CR before disease induction protects against EAE by improving T-cell function.

### Concept 2: CR enhances glucocorticoid production

Glucocorticoids have inhibitory effects on the expression of genes associated with inflammation [15]. Systemic administration of corticosteroids exacerbates MS [19]. In rodents, treatment with exogenous glucocorticoids blocks EAE [20]. In the study by Piccio et al., 4-week CR increased corticosterone levels [18]. Interestingly, endogenous production of corticosterone avoids the side-effects reported in the exogenous administration of corticosteroids [18].

### Concept 3: imposition of an immunomodulatory effect

Interleukin 6 (IL)-6 can be produced by adipose tissue. Four weeks of CR has been shown to reduce body fat and, consequently, IL-6 levels. IL-6 has a critical role in EAE induction [18]. IL-6 together with transforming growth factor (TGF)-β has been reported to inhibit the production of T regulatory cells, and results in suppression of expression of T-helper (Th)-17 genes [18]. Expression of Th-17 is one of the main features of MS [21].

### Fasting in human studies

There is limited evidence about fasting (especially Islamic fasting and MS). However, the immunomodulatory and antioxidative properties of fasting have been the subject of scientific investigations recently.

Ahmed T et al. undertook a study on 30% or 10% CR in 40 healthy overweight men and women over 6 weeks. They reported that delayed-type hypersensitivity responses (which reflect cell-mediated immunity) were increased significantly in both groups. Also, 30% CR reduced prostaglandin (PG) E2production and T-cell proliferative responses to anti-CD3. Moreover, they observed that proliferative T-cell responses to T-cell receptor antibodies and T-cell mitogens were increased in both groups. They concluded that CR can also improve T-cell function in humans [22]. Paliet al. compared the immune function of 12 obese adolescents with healthy, normal-weight adolescents. They found that the IFN- γ/IL-4 ratio in CD4+ cells was higher in obese individuals. They proposed that the antigen presenting cell-regulatory T-cell–CD4+ lymphocyte axis might be affected by calorie disturbance [23]. The effect of CR on humans has been studied in a randomized controlled trial sponsored by the USA National Institute on Aging. Preliminary results showed that 20–25% CR for 6–12 months reduces levels of leptin, T3 and fasting insulin. CR also reduces DNA damage as a marker of oxidative insult [24]. Fraser et al. conducted studies on the effect of fasting on patients with rheumatoid arthritis. Rheumatoid arthritis is an autoimmune disease that shares immunological features with MS. They reported that 7-day fasting reduced the activity of CD4+ lymphocytes and induced IL-4 production [25]. Latifynia et al. assessed the effect of Ramadan fasting on innate immunity (tetrazolium reaction in neutrophils and opsonization of phagocytes). They concluded that the innate immune response increases after fasting [26].

### Fasting and MS

Only two studies on Ramadan fasting in MS patients have been carried out. El-Dayem and Zyton carried out a study on 30 MS patients aged 15–45 years with an Expanded Disability Status Scale(EDSS) score <3. After 1 year, no significant differences were observed in relapse rate, EDSS score, and gadolinium-enhanced lesions on MRI between fasted and non-fasted groups [27]. Saadatnia et al. undertook a study on 80 patients aged >17 years with an EDSS score ≤3. After 6 months, no significant differences in EDSS score or frequency of clinical relapses were noted between fasted and non-fasted groups. El-Dayem and Zyton concluded that fasting does not have unfavorable effects in the short term. The authors also stated that reduction of food intake (especially of fat) during Ramadan could enhance antioxidative activity and consequently protect against relapse after Ramadan. El-Dayem and Zyton also suggested that reduction of levels of uric acid during Ramadan fasting could protect against MS relapse. Uric acid is formed as a metabolite of purine and is concentrated following dehydration during fasting. Serum levels of uric acid are lower in MS patients compared with healthy individuals [28].

In the section below, we present evidence (in the form of questions) from points discussed in the mini-symposium described at the start of this article.

### Question 1: Is it safe for MS patients to fast?

The effect of fasting on MS is not clear but Islamic fasting is, in general, considered to be safe in most MS patients. However, making a general recommendation about the safety of fasting in MS patients is not possible. The decision should be made with respect to individual conditions. Ramadan fasting does not appear to have negative effects on MS patients with mild disability in the short term [27,28]. Patients should be monitored for individual symptoms (e.g., fatigue, energy level), type of MS, level of disability, systemic disease, and medications taken as well as social skills. Patient education is an important part of management: they should be informed about the symptoms associated with exacerbation of MS.

### Question 2: Does fasting affect MS symptoms?

One of the main concerns is whether fasting affects MS symptoms. Authors have reported no short-term unfavorable effects of fasting on disease course [27,28]. However; it is believed that during fasting, some of the symptoms of MS might worsen but return to usual levels during feasting. Symptoms include: fatigue, dizziness, spasticity, cognitive problems, weakness, vision, balance, and gait.

Studies in healthy adults have revealed fasting to induce fatigue as well as the perception of fatigue. In a study carried out to assess the effect of Ramadan fasting on muscle power and fatigue in healthy football players after 2 weeks and 4 weeks of fasting, muscle power was decreased and fatigue increased in the evening. Moreover, the perception of exertion and fatigue is higher during Ramadan [29]. It seems that perceived fatigue is less common in MS patients who spiritually believe in fasting. However, in patients who believe fasting to be an obligation, perceived fatigue might be more prevalent. Moreover, heavy workers complain about tiredness and dizziness during Ramadan fasting, which may be due to dehydration [30].

Studies suggest that fasting has no effect on the vision of healthy adults. In healthy, middle-aged volunteers, fasting does not affect values of intraocular pressure, refractive error or visual activity [31]. However, it is believed that, unlike healthy adults, fasting might affect vision in MS patients. In a study on judo athletes, Ramadan fasting had negative effects on postural control: unipodal and bipodal sway velocity was decreased [32].

Studies on the effects of Ramadan fasting on the mood profile have elicited mixed results. In a study on eight middle-distance runners, a 30-day fast negatively affected the mood profile [33]. In another study on 20 young football players, the mood profile did not show significant differences during and after Ramadan compared with before Ramadan [34].

In another study on healthy athletes, changes in cognitive function were studied during Ramadan. The cognition domains were psychomotor functions, vigilance, visual learning, verbal learning, memory, and executive function. Comparison of cognitive function in the morning with the afternoon showed a significant decline in psychomotor function, visual and verbal learning, and memory. The extent of the effect was greater during Ramadan [35].

### Question 3: Does fasting affect MS exacerbation?

Studies suggest that Ramadan fasting neither protects nor provokes MS attacks. However, more prospective studies are needed to follow clinical conditions after Ramadan.

### Question 4: Can symptomatic treatments be taken during fasting?

One of the other most frequently asked questions about fasting in MS patients is whether symptomatic treatments are possible during fasting. It is believed that, for some drugs, amending the drug regimen is possible during Ramadan by substituting oral agents with injections, or by prescribing slow-release or long-acting drugs once or twice at night. However, accurate distribution of drugs prescribed twice a day or three times a day between the morning meal (Suhoor) and evening meal (Iftar) is difficult. If dosing and the time span between doses are changed, these alterations might affectthe serum level of the drug and, consequently, its tolerability and efficacy [3]. Furthermore, drug–food interactions can result in delayed, reduced or increased bioavailability of a drug. Also, changes in circadian rhythms sleep disturbances, emotional stress and physical stress can influence drug pharmacokinetics. In one study, even changing the timing of a single dose of valproate (800 mg) during Ramadan increased the frequency of seizures significantly [36]. The problem becomes more notable in patients on polytherapy. Table 1 summarizes circadian variations in the pharmacodynamics and pharmacokinetics of commonly prescribed medications for MS, as well as their drug–food interactions.

**Table 1:** Circadian variations in pharmacodynamics and pharmacokinetics as well as drug–food interactions of commonly prescribed MS drugs

**Drug**	**Food–drug interaction**	**Recommendation**
Baclofen	No effect [37].	
Dantrolene	No effect [38].	
Pregabalin	Consuming with food decreased C_max_by 25–30% and delayedt_max_by ≈3h [39].	
Tizanidine	Simultaneous food intake with a tizanidinecapsule reduced C_max_and AUC_o–t_ by <20% and extendedt_max_ from 0.75 h to 1.5 h.	Extent of absorption is increased ≤20% relative to administration of the tizanidine capsule under fasted conditions.
	In contrast,simultaneous food consumption with a tizanidinetablet increased C_max_ and AUC_o–t_ by22.6% and 45.2%, respectively [40].	The dosage forms of the tablet and capsule were not bioequivalent if administered with food.
		Food increasedthe t_max_ and the extent of absorption for the tablet and capsule. However, the C_max_values of tizanidine achieved if administered with food were increased by 30% for the tablet but decreased by 20% for the capsule. Under fed conditions, the capsule was ≈80% bioavailable relative to the tablet.
		Using tizanidine together with caffeine is, in general, not recommended. Combining these medications may significantly increase the blood levels and effects of tizanidine.
Gabapentin	High-protein food increased the AUC and C_max_by 26% and 32%, respectively [41].	Take extended release tablet with evening meal. Swallow whole; do not chew, crush, or split.
	No significant effects on the rate or extent of absorption of the immediate-release tablet were noted. The rate and extent of absorption of the extended-release tablet was increased.	
Carbamazepine	Serum levels of carbamazepine may be increased if taken with food and/or grapefruit juice.	Extended-release tablets should be administered with meals; swallow whole, do not crush or chew.
SSRIs	Paroxetine: peak concentration is increased, but bioavailability is not significantly altered by food.	
	Sertraline: average peak serum levels may be increased if taken with food.	

AUC_o–t_, area under the curve from administration (0) to last observed concentration at (t), C_max_, maximum concentration, t_max_, time after administration of a drug when the maximum plasma concentration is reached.

It is believed that fasting is not feasible for patients on high doses of anti-spastic drugs or antiepileptic drugs as well as patients taking drugs more than twice a day. Similarly, fasting should be avoided if changing the drug has negative effects on the activities of daily living or level of disability. There is also a general consensus that amending the drug regimen should be started before Ramadan. In a study on 114 epileptic patients, amending the drug regimen according to the timing of Suhoor and Iftar did not prevent an increase in seizure frequency [42].

### Question 5: Can disease-modifying drugs be consumed during fasting?

The pharmacokinetics and pharmacodynamics of interferons and glatiramer acetate are not affected by food consumption. Also, it seems that the plasma drug concentration–time curve of interferons and glatiramer acetate are not affected by prolonged fasting [43,44]. Data on the effect of fasting on the pharmacokinetics and pharmacodynamics of fingolimod are not available. However, according to some neurologists, fasting is not recommended for patients who experience severe flu-like symptoms (fever, chills, sweating, muscle aches, fatigue) after injection of interferons and glatiramer acetate.

Fasting might affect the pharmacokinetics and pharmacodynamics of immunosuppressants. Also, drug–food interactions may result in the increased/reduced/delayed systemic availability of immunosuppressants. Table 2 details the effects of fasting on the pharmacokinetics and pharmacodynamics of immunosuppressants and manufacturer recommendations for their consumption [45]. Pharmacokinetic and pharmacodynamic reactions between cyclophosphamide and foods have not been reported. However, cyclophosphamide is excreted mainly in the urine, so limited access to liquids during fasting may have negative effects on drug levels [46].

**Table 2:** Effect of fasting on the pharmacokinetics and pharmacodynamics of immunosuppressants and manufacturer recommendations for their consumption

**Drug**	**Circadian variations in pharmacodynamics and pharmacokinetics**	**Manufacturer recommendations**
Azathioprine		Take after meals or in divided doses for reducing the risk of stomach upset.
Cyclophosphamide		On an empty stomach with a glass of water or juice or with food.
		Tablets are not scored and should not be cut or crushed. Do not take tablets at bedtime to minimize the risk of bladder irritation.
Methotrexate	Peak serum levels of methotrexate may be decreased if taken with food. Milk-rich foods may decrease methotrexate absorption.	On empty stomach with plenty of water.
		Limit or avoid caffeine intake. Avoid drinks (e.g., coffee, tea, cola), foods (e.g., chocolate) or diet pills that contain caffeine.

### Question 6: Is the level of disability (EDSS score) important if a patient decides to fast?

As mentioned above for the data of Saadatnia et al. and El-Dayem, fasting is known to be safe in patients with mild disability (EDSS score ≤3) [27,28]. Patients with higher levels of disability are less physically active. Consequently, they are more prone to constipation, infection of the upper urinary tract, bedsore, and diverticulitis. Dehydration during prolonged fasting could aggravate such symptoms. It is recommended that patients with an EDSS score ≥7 avoid fasting.

### Question 7: Does the type of MS interfere with the decision to fast?

It is believed that, regardless of the type of MS, patients with highly active disease should be prohibited from fasting. Also patients who experience attacks during or after Ramadan fasting should avoid fasting. During attacks, because of the high dose of corticosteroid therapy, fasting is not recommended.

### Question 8: Is fasting during summer safe for MS patients?

The demands of fasting at high latitudes during winter are different from those in equatorial regions during summer [47]. Increasing the number of fasting hours during summer can increase the negative effects of fasting. Individualized monitoring of MS patients who observe fasting during summer and avoiding dehydration is recommended.

### Question 9: Is it safe for MS patients to fast for a whole month?

The interval between fasting days should be tailored to the condition of the patient. Appropriate sleep, adequate and appropriate intake of food and fluids during feasting hours are recommended. According to Saadatnia et al., fasting for 13 h a day for 28 days does not result in unfavorable short-term effects [28].

## Summary

Fasting during Ramadan is an obligation for healthy Muslim adults. Studies on fasting in MS patients (especially Islamic fasting) are very rare and have focused on the protective role of intermittent fasting in MS patients. There is insufficient evidence to suggest fasting can protect against or control MS signs, but fasting does not have unfavorable effects on disease course.

In the mini-symposium discussed above, there is a general consensus that observation of Ramadan fasting is possible for many MS patients. Careful monitoring of general and clinical conditions as well as precise management of drug regimens, diet, and sleep patterns will help to reduce the potential negative effects of fasting. However, fasting is not recommended during attacks, in patients on high doses of anti-spastic and anti-convulsant drugs, or in those with coagulopathy, and EDSS score ≥7, or active disease.

Fasting (especially during summer) might negatively affect fatigue, weakness, vision, balance, and gait.Individualized recommendations should be made with respect to energy level and general wellbeing. It seems that MS patients who believe in fasting as a sacred obligation report fewer negative impacts of fasting on MS signs. For example, perceived fatigue is increased during fasting. Spiritual belief might reduce the perception of fatigue. This hypothesis might be expandable to other symptoms of the disease. Conversely, in patients who find themselves obliged to fast, the perception of the negative impacts of fasting might be greater. Patients should be provided with scientific evidence about the effects of fasting on health issues before Ramadan to make informed decisions about fasting.

More studies are needed to prepare detailed guidelines for MS patients. Our database search and mini-symposium meeting were attempts to help physicians for the better care of MS patients who decide to fast, as well as to pave the way for more research in this field.

## Abbreviations

AA: Acetoacetate; ADF: Alternate-day fasting; CNS: Central nervous system; CR: Calorie restriction; EAE: Experimental autoimmune encephalomyelitis; EDSS: Expanded disability status scale; HNE: 4-hydroxynonenal; KD: Ketogenic diet; IFN-γ: Interferon-gamma; IL-6: Interleukin-6; MDA: Malondialdehyde; MS: Multiple sclerosis; NF-κ B: Nuclear factor kappa B; Th-17: T-helper 17; TGF-β: Transforming growth factor beta; TNF-α: Tumor necrosis factor-α.

## Competing interests

None of the authors had any financial or personal conflicts of interest.

## Authors’ contributions

SRJ: Wrote the primary draft. MAS: Proposed the idea, managed the meeting, and revised the primary draft. FA: Active participation in the meeting, reviewing the literature and approving the primary draft. HA: Active participation in the meeting, reviewing the literature and approving the primary draft. ME: Active participation in the meeting, reviewing the literature and approving the primary draft. MG: Active participation in the meeting, reviewing the literature and approving the primary draft. EM: Active participation in the meeting, reviewing the literature and approving the primary draft. SN: Active participation in the meeting, reviewing the literature and approving the primary draft. AN: Active participation in the meeting, reviewing the literature and approving the primary draft. VS: Active participation in the meeting, reviewing the literature and approving the primary draft. MT: Active participation in the meeting, reviewing the literature and approving the primary draft. HRT: Active participation in the meeting, reviewing the literature and approving the primary draft. SZ: Active participation in the meeting, reviewing the literature and approving the primary draft. All authors read and approved the final manuscript.

## Pre-publication history

The pre-publication history for this paper can be accessed here:

http://www.biomedcentral.com/1471-2377/14/56/prepub
